# Maternal overnutrition by hypercaloric diets programs hypothalamic mitochondrial fusion and metabolic dysfunction in rat male offspring

**DOI:** 10.1186/s12986-018-0279-6

**Published:** 2018-06-05

**Authors:** Robbi E. Cardenas-Perez, Lizeth Fuentes-Mera, Ana Laura de la Garza, Ivan Torre-Villalvazo, Luis A. Reyes-Castro, Humberto Rodriguez-Rocha, Aracely Garcia-Garcia, Juan Carlos Corona-Castillo, Armando R. Tovar, Elena Zambrano, Rocio Ortiz-Lopez, Jennifer Saville, Maria Fuller, Alberto Camacho

**Affiliations:** 1 Departmento de Bioquímica y Medicina Molecular, Facultad de Medicina, Universidad Autonoma de Nuevo Leon, Monterrey, Mexico; 2 Unidad de Neurometabolismo, Centro de Investigación y Desarrollo en Ciencias de la Salud, Universidad Autónoma de Nuevo Leon, Monterrey, Mexico; 3 Centro de Investigacion en Nutricion y Salud Publica, Facultad de Salud Publica y Nutricion, Universidad Autonoma de Nuevo Leon, Monterrey, Mexico; 4 Departamento Fisiología de la Nutrición, Instituto Nacional de Ciencias Medicas y Nutrición, Mexico City, Mexico; 5 Departamento de Biología de la Reproducción, Instituto Nacional de Ciencias Medicas y Nutrición Salvador Zubiran, México City, Mexico; 6 Departmento de Histología, Facultad de Medicina, Universidad Autonoma de Nuevo Leon, Monterrey, Mexico; 7 Laboratorio de Neurociencias, Hospital Infantil de México, Federico Gómez, México City, Mexico; 8 Escuela de Medicina y Ciencias de la Salud, Instituto Tecnologico de Monterrey, Monterrey, Mexico; 9 Genetics and Molecular Pathology, SA Pathology at Women’s and Children’s Hospital, University of Adelaide, Adelaide, Australia; 10 Departamento de Bioquimica y Medicina Molecular. Facultad de Medicina, Universidad Autónoma de Nuevo León, Ave. Francisco I Madero y Dr. Eduardo Aguirre Pequeño s/n. Colonia Mitras Centro, C.P. 64460 Monterrey, Nuevo Leon Mexico

**Keywords:** Maternal overnutrition, Diet induced obesity (DIO), Hypothalamus, Mitochondria, Mitochondria dynamics, Fusion, Fission

## Abstract

**Background:**

Maternal overnutrition including pre-pregnancy, pregnancy and lactation promotes a lipotoxic insult leading to metabolic dysfunction in offspring. Diet-induced obesity models (DIO) show that changes in hypothalamic mitochondria fusion and fission dynamics modulate metabolic dysfunction. Using three selective diet formula including a High fat diet (HFD), Cafeteria (CAF) and High Sugar Diet (HSD), we hypothesized that maternal diets exposure program leads to selective changes in hypothalamic mitochondria fusion and fission dynamics in male offspring leading to metabolic dysfunction which is exacerbated by a second exposure after weaning.

**Methods:**

We exposed female Wistar rats to nutritional programming including Chow, HFD, CAF, or HSD for 9 weeks (pre-mating, mating, pregnancy and lactation) or to the same diets to offspring after weaning. We determined body weight, food intake and metabolic parameters in the offspring from 21 to 60 days old. Hypothalamus was dissected at 60 days old to determine mitochondria-ER interaction markers by mRNA expression and western blot and morphology by transmission electron microscopy (TEM). Mitochondrial-ER function was analyzed by confocal microscopy using hypothalamic cell line mHypoA-CLU192.

**Results:**

Maternal programming by HFD and CAF leads to failure in glucose, leptin and insulin sensitivity and fat accumulation. Additionally, HFD and CAF programming promote mitochondrial fusion by increasing the expression of MFN2 and decreasing DRP1, respectively. Further, TEM analysis confirms that CAF exposure after programing leads to an increase in mitochondria fusion and enhanced mitochondrial-ER interaction, which partially correlates with metabolic dysfunction and fat accumulation in the HFD and CAF groups. Finally, we identified that lipotoxic palmitic acid stimulus in hypothalamic cells increases Ca^2+^ overload into mitochondria matrix leading to mitochondrial dysfunction.

**Conclusions:**

We concluded that maternal programming by HFD induces hypothalamic mitochondria fusion, metabolic dysfunction and fat accumulation in male offspring, which is exacerbated by HFD or CAF exposure after weaning, potentially due to mitochondria calcium overflux.

## Background

Maternal obesity in humans associates with an increased risk of obesity metabolic-related disorders in offspring [1, 2]. It is known that obesity and maternal overnutrition create changes in uterine milieu during pregnancy leading to developmental alterations and defects in organ function and metabolism in offspring [3, 4]. Also, maternal nutritional programming by hypercaloric diets exposure that simulate Western diets, such as high fat diet (HFD), cafeteria diet (CAF) or high sugar diet (HSD) in murine models, change offspring metabolism leading to insulin resistance, type 2 diabetes mellitus (T2DM) [5–7], cardiovascular diseases and hypertension [8], non-alcoholic liver diseases and steatohepatitis [9, 10]. Defective molecular pathways related to maternal programming by nutrient oversupply can lead to failure in mitochondria dynamics including modifications in mitochondria fission and fusion, which are potentially linked to metabolic compromise and disease susceptibility [11].

Diet-induced obesity murine models alter mitochondrial function and dynamics in selective organs including muscles, adipose tissue, liver and brain [11]. In particular, maternal obesity in rodents is associated with altered mitochondria function, reactive oxygen species generation and an increased mtDNA and mitochondrial biogenesis in oocytes and zygotes [12]. Likewise, oocytes and blastocysts from obese mice show reduced mitochondrial membrane potential, high levels of autophagy and reduced mtDNA and mitochondrial biogenesis [13, 14], potentially linked to hepatic lipotoxic insult in fetuses of obese females [13]. In fact, mitochondrial function in offspring after maternal programming seems to be sex-dependent, showing insulin resistant and oxidative stress in males in compare with females [15, 16]. Finally, in humans, placentas of obese mothers show a decrease in mtDNA and mitochondrial dysfunction which correlate with metabolic dysfunction in offspring [17].

Mitochondrial functions are modulated by fission and fusion dynamic processes which assist to maintaining mitochondria homeostasis [18]. Mitofusin 1 and 2 (MFN 1 and 2) and OPA1 and DRP1 modulate fusion and fission processes, respectively, helping to reduce cellular stress by joining mitochondria or creating new mitochondria to assists deficient mitochondria [18]. Maternal programming by HFD exposure in rodent dams, decrease expression of OPA1 and DRP1 proteins in skeletal muscles of female offspring and disrupt mitochondrial function in male offspring [19, 20], which might be transmitted across three generations [20]. Furthermore, we and others have reported that obese mice showed changes in MFN2 expression in hypothalamus and other tissues [21–24], and importantly mitochondria elongate in a Mfn1/2 dependent manner in Agouti-related peptide neurons [25]. Here, we hypothesized that maternal nutritional programming before and after weaning by selective diets might modulate mitochondria fusion and fission dynamics in male offspring leading to metabolic dysfunction.

## Methods

### Animals, diets and nutritional programming model in offspring by HFD, CAF and HSD exposure

**Fig. 1:**
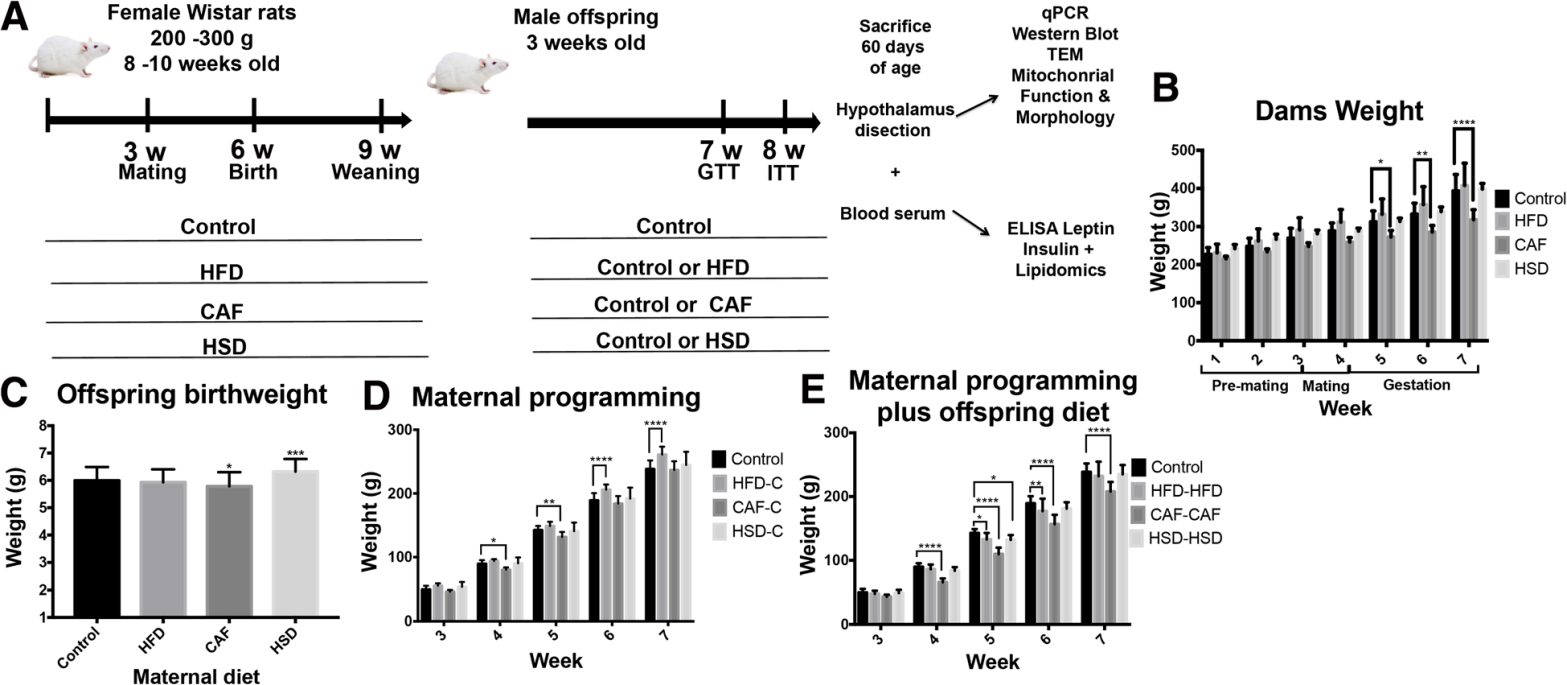
Effect of nutritional programming on dams and male offspring weight. **a**) Animal model. We fed female Wistar rats for 9 weeks according to the schedule to promote maternal programming or we fed offspring with hypercaloric diet exposure after weaning. **b**) Maternal body weight was followed for 6 weeks (pre-mating, mating and gestation) (Control *n* = 7, HFD n = 7, CAF *n* = 6 and HSD *n* = 4). **c**) Birth weight of offspring at day 0. **d**) Body weight after weaning for Control versus HFD-C, CAF-C, HSD-C for maternal programming groups, where HFD, CAF and HSD are maternal diet and C is control chow diet of offspring. **e**) Body weight after weaning for Control versus offspring fed with HFD, CAF and HSD after weaning (HFD-HFD, CAF-CAF, HSD-HSD groups). Data are means ± SD. *p < 0.05, **p < 0.01 and ***p < 0.001

All the experiments were first performed using 8–10 weeks-old female Wistar rats, 200–250 g (*n* = 24). Animals were handled according to the NIH guide for the care and use of laboratory animals (NIH Publications No. 80–23, revised in 1996), with approval of the local Animal Care Committee (BI0002). All efforts were made to minimize the number of animals used and their suffering. Rats were housed individually in Plexiglas style cages, maintained at 22–23 °C and 12-h light/dark cycle. Water and food was available ad libitum. Animals were acclimated to the animal facility 7 days prior to diets exposure. Females rats were randomized into four dietary groups: Control diet, HFD diet, CAF diet and HSD diet (Table 1). The formulation of diets was: Control that contained a caloric density of 3.35 kcal/g divided in 71% carbohydrates, 11% lipids and 18% proteins (Research Diets, New Brunswick, NJ, Cat. D12450B). High-fat diet (HFD) contained a caloric density of 4.9 Kcal/g divided in 45% lipids, 20% proteins and, 35% carbohydrates. Cafeteria (CAF) diet was made of liquid chocolate, biscuits, bacon, fries potatoes, standard diet and pork pate based on a 1:1:1:1:1:1:2 ratio, respectively; total calories 3.72 kcal/g in 39% carbohydrates, 49% lipids, 12% proteins and 513.53 mg of Sodium. High sugar diet (HSD) composition was Standard diet and condensed milk on a 1:1.5 ratio, respectively; total calories 3.39 kcal/g in 69% carbohydrates, 18.5% lipids, 12.5% proteins and 228 mg of Sodium. It is important to point out that these diets appear to be found in human population. After randomization, female rats were fed for 9 weeks, including 3 weeks of pre-mating, mating, birth, and lactation. Rats were mated with 12–14 weeks old Wistar males, 300–350 g, during two days. Also, it is important to highlight that body weight was quantified in these females every week during pre-mating and gestation (See Fig. 1a). We registered body weight of all offspring at birth (approximately 15 rats/litter) and at the age of 3 weeks we euthanized female offspring. Male offspring was grouped into 10–12 subjects per group and were allocated into two groups: Group 1) exposure to Chow control diet to analyze maternal programming, including three groups: Maternal HFD and offspring Chow control diet (HFD-C), Maternal CAF diet and offspring Chow control diet (CAF-C) and Maternal HSD and offspring Chow control diet (HSD-C); Group 2) exposure to the same diet of their mothers until the age of 8 weeks, resulting in three groups: Maternal HFD and offspring HFD (HFD-HFD), Maternal CAF diet and offspring CAF diet (CAF-CAF) and Maternal HSD and offspring HSD (HSD-HSD). We compared these groups with a group where females and their offspring were fed with Chow Control diet. A total of seven experimental groups were included: Control, HFD-C, CAF-C, HSD-C, and HFD-HFD, CAF-CAF and HSD-HSD. Body weight, food and calorie intake were quantified in offspring from 3rd-7th week following by metabolic assessments as described below (see Fig. 1a for details). The daily calorie intake was calculated from the weight of food consumed multiplied by the calories/gram of the food.

**Table 1:** Diet Composition

Diet	Percentage of macronutrients	Ingredients
Control	Carbohydrates 71% Lipids 11% Proteins 18% Caloric density 3.35 kcal/g	Cystine 0.14 g, Coline 0.19 g, Vitamins 1.89 g, Celulose 2.89 g, Minerals 4.73 g, Soybean oil 5.16 g, Starch 32.59 g, Dextrin 16.60 g, Sucrose 16 g, Casein 19.87 g per 100 g
HFD	Carbohydrates 35% Lipids 45% Proteins 20% Caloric density 4.9 kcal/g	Cystine 0.17 g, Coline 0.23 g, Vitamins 2.99 g, Celulose 1.72 g, Minerals 5.57 g, Inulin 1.72, Soybean oil 5.75 g, Dextrin 29.46 g, Sucrose 10.35 g, Casein 24.14 g, Lard 17.72 g per 100 g
CAF	Carbohydrates 39% Lipids 49% Proteins 12% Caloric density 3.72 kcal/g	Chow diet 14.29 g, liquid chocolate 14.29 g, biscuits 14.29 g, bacon 14.29 g, fries potatoes 14.29 g, pork pate 28.58 g 1:1:1:1:1:1:2 ratio per 100 g
HSD	Carbohydrates 69% Lipids 18.5% Proteins 12.5% Caloric density 3.39 kcal/g	Chow diet 40 g, condensed milk 60 g 1:1.5 ratio per 100 g

### Glucose tolerance and insulin tolerance test (GTT, ITT) assessments

To test if maternal programming by selective hypercaloric diets leads to alterations in the tolerance of glucose and insulin, the GTT and ITT tests were carried out in the offspring. Males were 8 h–12 h fasted and then were intraperitoneally injected with 40% glucose body weight or 1 U of insulin/100 g body weight. Blood glucose levels were quantified at 0 min, 15 min, 30 min, 45 min, 60 min, 90 min, and 120 min, as described previously [26]. These tests were performed at the age of 7 weeks for GTT and 8 weeks for ITT in each male rat.

### Tissue samples collection

Male rats were sacrificed by decapitation at 9 weeks of age. Blood samples were collected in 500 μL tubes (Beckton Dickinson) and plasma fraction was isolated by centrifugation at 4 °C and frozen at − 80 °C. Hypothalamus was dissected and divided into the left hemisphere (for RNA extraction and gene expression analysis) and right hemisphere (frozen immediately at 80 °C for western blot analysis). Also, liver and retroperitoneal white adipose tissue was measured and collected.

### Plasma biochemistry determination

Glucose was determined by glucose strips and Accu-check ® (Roche, Cat. 05987270), insulin (Millipore Inc., Cat. EZRMI-13 k) and leptin (Millipore Inc., Cat. EZML-82 K) were determined by Elisa kits according to manufacturers’ instructions.

### Mass spectrometric determinations of triglycerides

Triglycerides were extracted from 10 μL of plasma using a single phase extraction method as described in Fuller et al. (2015), with the exception that 100 pmol of glyceryl triheptadecanoate (TG (17:0/17:0/17:0); Sigma-Aldrich, Cat. T2151) was added to each sample as the internal standard. Extracted triglycerides were first separated on a RRHD Eclipse Plus C18 column (2.1 × 150 mm; 1.8 μm) maintained at 40 °C. The samples were maintained at 16 °C with 3 μL injected into the mobile phase which was at a flow rate of 0.4 mL/min. Mobile phase A consisted of 60% H_2_O, 40% CH_3_CN containing 10 mM NH_4_COOH and solvent B was 90% (CH_3_)_2_CHOH, 10% CH_3_CN containing 10 mM NH_4_COOH. Mobile phase conditions were 90% solvent A and 10% solvent B at injection, which was linearly ramped to 50% by 6 min and then to 100% solvent B at 24 min. This was maintained for 3 min before returning to 90% solvent A at 28 min, where the column equilibrated for 4 min prior to the next injection.

For the first 4 min column flow was diverted to waste before being directed into the electrospray source (ES 5500 V) of an AB SCIEX QTRAP 6500 triple quadrupole tandem mass spectrometer with an ion source temperature of 250 °C. Nitrogen was used for curtain gas, 25 units; collision gas set at medium; nebulizer gas 1, 20 units and auxiliary gas 2, 40 units. Declustering potential was 120 V; entrance potential, 8 V, collision energy, 36 V and collision exit potential, 26 V. Individual species of triglycerides were measured by multiple reaction monitoring in positive ion mode using the ammonium adduct and corresponding fragment arising from the neutral loss of one fatty acid. As such, only the sum composition (i.e. total number of carbons and double bonds) of the remaining two remaining fatty acyl chains could be determined. Sixty three transitions were monitored with 57 species detected in plasma. Fatty acyl chain lengths ranged from 14:0 to 22:6 and of particular interest were the following transitions: 16:0/36:4 (872.8/599.6), 18:0/34:1 (878.8/577.5), 18:2/36:2 (900.9/603.6) and 18:0/36:2 (904.9/603.6). Six species were present at high concentrations, requiring quantification from the [M + 1] isotopologue due to saturation of the detector by the mono-isotopic ion. These were 16:1/34:2 (847.8/576.6), 18:1/32:1 (849.8/550.5), 18:1/32:0 (851.8/552.6), 18:2/34:1 (875.8/578.6), 16:0/36:2 (877.8/604.6) and 18:1/36:2 (903.9/604.6). The concentration of each species was calculated using MultiQuant 3.0.1 software (AB SCIEX, Framingham, MA, USA) by relating the peak area of each species to that of the internal standard.

### Western blot analysis

Frozen hypothalamus was homogenized in 500 μl lysis buffer as described previously with minor modifications [27]. Samples were subjected to SDS–PAGE, nitrocellulose membranes blocked for 2 h at RT in TBS-T buffer (10 mM Tris, 0.9% NaCl, 0.1% Tween 20, pH 7.5) containing 5% BSA (Santa Cruz Biotecnology, Inc., cat. sc2323) and incubated overnight with primary antibodies at 4 °C in TBS-T Buffer 1% BSA: anti-MFN2 (Abcam, Product code ab56889, 1:1000), anti-Drp1 (Cell Signaling, Cat. 8570, Dilution 1:2000) and B-actin (Cell Signaling, Cat. 8457, Dilution 1:3000). Anti-mouse and anti-rabbit horseradish Peroxidase-conjugated were used as secondary antibody in TBS-Buffer 5% BSA (Cell Signaling, Cat. 7076S & Cat. 7074P29, Dilution 1:3000). Proteins were detected by chemiluminescence in the Chemidoc XRS+ System (BioRad) using Clarity Western ECL Blotting Substrates (BioRad, Cat. No. 1705061). Images were quantified densitometrically with ImageJ Software 1.50i (Wayne Rasband, National Institutes of Health, Bethesda, MD, USA).

### RNA isolation and real time (RT)-PCR

RNA extraction from hypothalamic samples was performed as described previously [28]. RT-PCR was performed by High-Capacity cDNA Reverse Transcription Kit (Applied biosystems, Cat. 4,368,814) using random primers and following standardized protocols.

### Quantitative PCR

Based on that, obesity modulates ER stress and mitochondria fusion and fission dynamics markers in animal models, we identified changes in mRNA of these markers in the offspring exposed to maternal programming. We performed a quantitative PCR using cDNA (10 ng), Light Cycler SBYR green 480 Master Mix (Roche LifeScience, Product No. 04707516001) and the following primers: MFN2 Forward (5’-CCATGTGTCGCTTATCCTTCT-3′), Reverse (5’-TGACTCCAGCCATGTCCAT-3′); Itpr Forward (5’-CACCTATGACCACACTGTCTC-3′), Reverse (5’-AAGAACFCCATGAGAGTGAC-3′); Hspa5 Forward (5’-CCAGTCAGATCAAATGTACCCA-3′), Reverse (5’-ATCAGCCCACCGTAACAATC-3′); Tfam Forward (5’-GTACACCTTCCACTCAGCTTT-3′) Reverse (5’AGCTAAACACCCAGATGCAA-3′); Drp1 Forward (5’-AACCCTTCCCATCAATACATCC-3′) Reverse (5’-TCCAGAGAGGTAGATCCAGATG-3′) and GAPDH like endogenous gen Forward (5’-GTAACCAGGCGTCCGATAC-3′), Reverse (5’-TCTCTGCTCCTCCCTGTTC-3′) (Integrated DNA Techologies, Inc.) in LightCycler ® 480 Instrument II (Roche LifeScience, Product No. 05015278001).

### Transmission electron microscopy

To elucidate changes in mitochondrial and ER morphology in the hypothalamus linked to hypercaloric diets exposure, a TEM study was performed. Tissue samples of hypothalamus of CAF-CAF and Control groups (*n* = 4) were fixed with 2.5% glutaraldehyde in 0.1 M sodium cacodylate (pH 7.4) for 2 h at room temperature, post-fixed with 1% OsO4 in 0.1 M sodium cacodylate, and counterstained in 1% uranyl nitrate. Tissue samples were dehydrated through a graduated acetone series and embedded in Epon 812 resin for sectioning. Ultrastructural images of thin sections were observed under a transmission electron microscope Carl-Zeiss EM 109, and collected with a bottom-mount film-based camera.

### Measurements of mitochondrial mass, membrane potential (ΔΨm) and ER activity

Obesity favours a lipotoxic environment with palmitic acid being one of the lipids related to this response and known to increase ER stress and mitochondrial dysfunction. To evaluate the effects of lipotoxicity on the mitochondria of the hypothalamus, we used an in vitro model of hypothalamic cells. Hypothalamic mHypoA-CLU192 cells were grown on 22 mm coverslips in growth medium (1× DMEM with 10% fetal bovine serum, FBS), 25 mM glucose and 1% penicillin/streptomycin) and maintained at 37 °C with 5% CO2 for 24 h. Cells were pre-treated with indicated treatments and then cells were loaded with recording medium (RM) consisting of; 156 mM NaCl; 3 mM KCl; 2 mM MgSO4; 1.25 mM KH2PO4; 10 mM D-Glucose; 2 mM CaCl2; 10 mM Hepes; with pH 7.3–7.4, containing 25 nM TMRM (Tetramethylrhodamine, Molecular Probes, Invitrogen Cat. T668) for ΔΨm and 1 μM ER-Tracker Green (Molecular Probes, Invitrogen, Cat. E34251) for 30 min at room temperature (RT) and were washed with saline solution containing 25 nM TMRM. Images were acquired using a Zeiss Axiovert 100 M confocal microscope with a Plan-Neofluar × 63/1.25 oil immersion objective lens at RT. TMRM fluorescence was excited at 543 nm and ER-Tracker Green excited at 488 nm wavelength laser. Images were analysed using the software Image J. For each plane of the z-stack, the two channels ER-Tracker Green and TMRM were separated, following the subtraction of the background fluorescence (i.e. threshold), to acquire two separate binary images. The proportion of the cytoplasm (stained with ER-Tracker Green) occupied by the mitochondrial network (stained with TMRM) was then calculated from the area of both images, calculating mitochondrial volume fraction occupancy of the cytosol. Images were analysed using the software program Image J.

### Mitochondrial Ca^2+^ levels

We assessed if lipotoxicity induced by palmitic acid promotes failure in mitochondrial calcium homeostasis and mitochondrial dysfunction linked to calcium-overload. Cells were grown on 22 mm coverslips and pre-treated with indicated treatments and then were loaded with RM consisting of: 156 mM NaCl; 3 mM KCl; 2 mM MgSO4; 1.25 mM KH2PO4; 10 mM D-Glucose; 2 mM CaCl2; 10 mM Hepes; with pH 7.3–7.4, containing 20 μM Rhod-2 (Molecular Probes, Invitrogen, Cat. R1245), AM and 0.02% Pluronic F-127 for 30 min at RT. The fluorescence intensity was determined every 15 s. Images were obtained using a Zeiss Axiovert 100 M confocal microscope with a Plan-Neofluar × 63/1.25 oil immersion objective lens and equipped with a helium-neon laser at RT. Fluorescence images labelled with Rhod-2 AM were collected using an excitation wavelength of 514 nm. Rhod-2, AM fluorescence was normalized and plotted using the software Image J.

### Statistical analysis

Statistical analysis was performed with GraphPad Prism Software (Version 7.0a Graph Pad Software Inc., La Jolla, CA). Data were expressed as Mean ± SD. The data presented were analyzed using Two-way analysis of variance (Two-way ANOVA), analysis of variance (ANOVA) or Student’s t-test with post-hoc test of Dunnett’s multiple comparison test, *p < 0.05, **p < 0.01, ***p < 0.001, ****p < 0.0001 were considered significant.

## Results

### Selective nutritional programming alters pregnancy ratio, body weight and food intake in offspring

Our aim was to evaluate the effect of nutritional programming by maternal hypercaloric diets in mitochondrial dynamics in the hypothalamus of the offspring and evaluate if this effect is exacerbated when offspring is exposed to these formula after weaning. We determined the effect of hypercaloric diets on body weight of female Wistar rats pre-mating, mating and during gestation (Fig. 1b). Female rats were fed with hypercaloric formula (HFD, CAF or HSD) do not modify body weight before mating; however, there was a significant decrease in female body weight during CAF exposure at 5–7 weeks and there were no changes during HFD and HSD exposure (Fig. 1b). While alterations in insulin and leptin levels have been found in mothers fed hypercaloric diets during pregnancy and lactation [29–31], it is important to point out that in our murine model, maternal hypercaloric diet intake does not develop obesity in mothers. Our main aim at this stage was to identify metabolic changes in male offspring linked to maternal over nutrition. To address this aim, pups (male and female) from mothers were randomized at the age of 3 weeks in 7 experimental groups as previously described. Initially, we found that nutritional programming by CAF diet decreased offspring weight whereas HSD litters increases their body weight at birth (Fig. 1c). Next, we assessed weight during 6–7 weeks of age of male offspring. The HFD-C group showed an increase in body weight at 6–7 weeks old when compared to control group, similar to their mothers (Fig. 1d). Offspring exposed to CAF diet programming (CAF-C) showed a decreased in total body weight at age of 4–5 weeks old and caught up control body weight at 6 weeks (Fig. 1d). Also, hypercaloric challenged after weaning showing in the HFD-HFD offspring group, decreased weight at 5–6 weeks and caught up control body weight at 7 weeks (Fig. 1e). Moreover, CAF-CAF diet exposure decreased body weight from week 4 to week 7 of age and they did not recover to control values (Fig. 1e). Finally, during the HSD-HSD exposure we do find a significant decrease in body weight at week 5 which caught up to control values at week 6 (Fig. 1e).

**Fig. 2:**
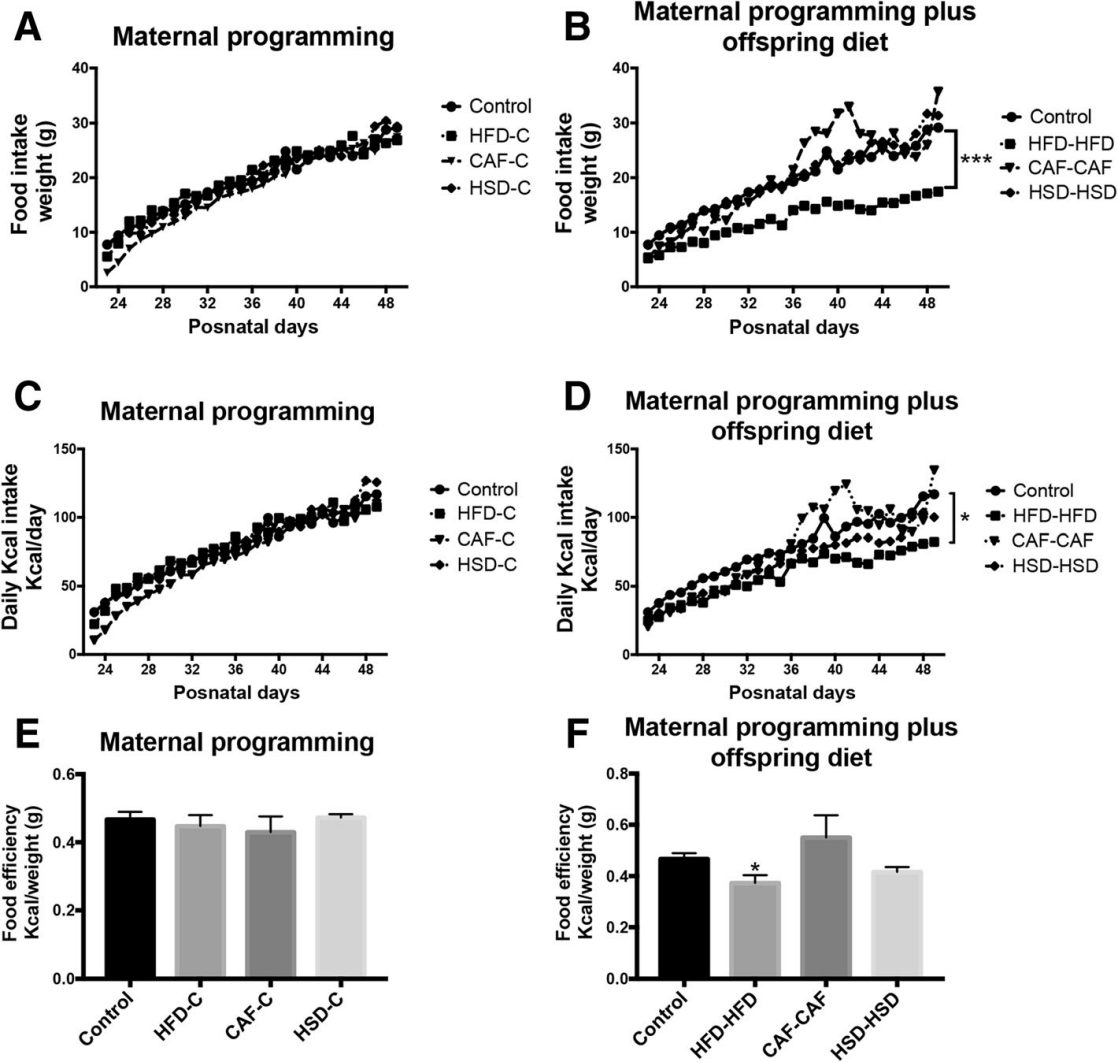
Effect of nutritional programming and hypercaloric diet exposure after programming (offspring diet) on food consumption. **a** and **d**) Food was weighted daily for 28 days for the different groups both maternal programming and offspring diet. **b** and **e**) Kcal per day was calculated by group in this time frame. **c** and **f**) Food efficiency by week was calculated. Data are means ± SD. *p ≤ 0.05

Next, we quantified the total food intake and body weight every day in male offspring linked to diets exposure between 23 to 49 days of age. Subjects from the HFD-C, CAF-C and HSD-C groups did not show change in food intake consumption or total kcal/day intake (Fig. 2a). By contrast, hypercaloric diet exposure after programming in the HFD-HFD group showed a decrease in food intake and had less kcal/day intake when it was compared to Chow control group (Fig. 2b), this contrast with their weight where we did not found difference in compare with control group at age of 7 weeks (Fig. 2b, c, d and Fig. 1e). Worthy of note, the CAF-CAF group had hyperphagic behavior from day 36 to day 45 of age and decreased weight when compared with control group from 4 week until 7 week of age (Fig. 2b, c, d and Fig. 1e). Lastly, we evaluated food efficiency by showing the kcal/weight ratio and observed that HFD maternal programming does not affect food efficiency (Fig. 2e); however, fat hypercaloric surplus after programming in the HFD-HFD group, displayed a significantly decrease in food efficiency (Fig. 2f). These results suggest that maternal programming by HFD and CAF exposure led to failure in their weight and energy homeostasis, and these alterations were exacerbated in their offspring fed with HFD and CAF diets after weaning.

### Cafeteria diet exposure during pregnancy and lactation disrupts glucose sensitivity in male offspring

**Fig. 3:**
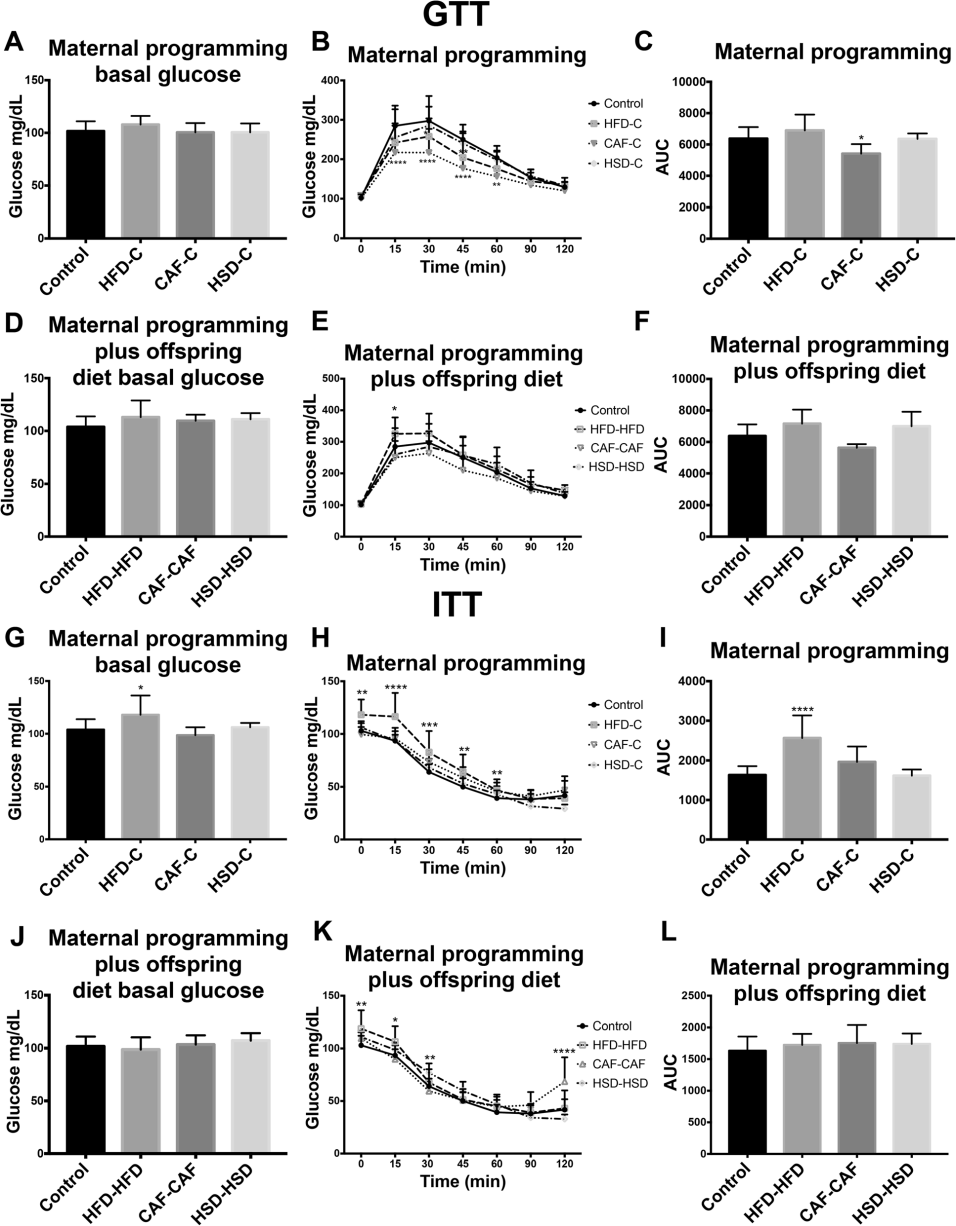
Effect of nutritional programming and offspring diet on glucose homeostasis. **a**, **d**, **g** and **j**) Basal glucose was measured for all groups. **b**, **e**, **h** and **k**) GTT and ITT Tests were performed at 15, 30, 45, 60, 90, and 120 min. **c** and **f**) Area Under Curve for GTT. **i** and **l**) Area Under Curve for ITT. Data are means ± SD. *p < 0.05, **p < 0.01 and ***p < 0.001

Male offspring tolerance to glucose and insulin was analyzed at 7 and 8 weeks old by performing GTT and ITT, respectively, to determine whether hypercaloric diets disrupt glucose sensitivity. Initially, we found no significant differences in basal glucose levels in the seven experimental groups during GTT test (Fig. 3a, d). Additionally, there were no changes in glucose sensitivity evaluated during the GTT in maternal programming groups (HFD-C, HSD-C) and in the offspring hypercaloric exposure after programming (HFD-HFD, HSD-HSD and CAF-CAF) (Fig. 3b, c, e, f). Besides, nutritional programing by CAF-C exposure decreased plasma glucose concentration and showed a significant decrease in AUC (area under the curve) during the GTT (Fig. 3b, c). Next, during the ITT the HFD-C group displayed an increase in basal glucose plasma levels when compared to Chow control group (Fig. 3g). Also, ITT showed reduced insulin sensitivity in HFD-C and CAF-C groups during versus Control (Fig. 3h, i) and significant increase in AUC for HFD-C group (Fig. 3i). There were no changes in insulin sensitivity in HSD-C, CAF-CAF and HSD-HSD groups (Fig. 3j, k, l). These results suggest that maternal programing by HFD and CAF exposure might affect glucose homeostasis in male young offspring.

Afterwards, we determined leptin and insulin plasma levels associated to maternal diet and hypercaloric diets in offspring after weaning. We found that maternal programming by HFD and CAF or HSD (HFD-C and CAF-C or HSD-C) increase plasma insulin and leptin concentration, respectively (Table 2). Also, the plasma insulin and leptin concentration were found increased in the groups HFD-HFD and CAF-CAF when they were compared to Chow control group (Table 2).

**Table 2:** Concentrations of insulin and leptin

Insulin				
Experiment	Group	Concentration ng/μL	SD	*P* value
Maternal programming	Control	0.0782	0.0033	N/A
	HFD-C	0.163	0.055	0.0009***
	CAF-C	1.234	0.4971	0.6189
	HSD-C	0.074	0.0142	0.9617
Maternal programming plus diet offspring	Control	0.0782	0.0033	N/A
	HFD-HFD	0.2478	0.0808	0.0001****
	CAF-CAF	0.2644	0.0542	0.0001****
	HSD-HSD	0.08941	0.0071	0.951
Leptin				
Experiment	Group	Concentration ng/μL	SD	*P* value
Maternal programming	Control	0.0671	0.0191	N/A
	HFD-C	0.063	0.0099	0.7709
	CAF-C	17.026	3.983	0.0001****
	HSD-C	0.0526	0.0031	0.0355*
Maternal programming plus diet offspring	Control	0.0671	0.0191	N/A
	HFD-HFD	0.1036	0.0189	0.0007***
	CAF-CAF	0.09779	0.0202	0.0002***
	HSD-HSD	0.05895	0.00496	0.5173

**Fig. 4:**
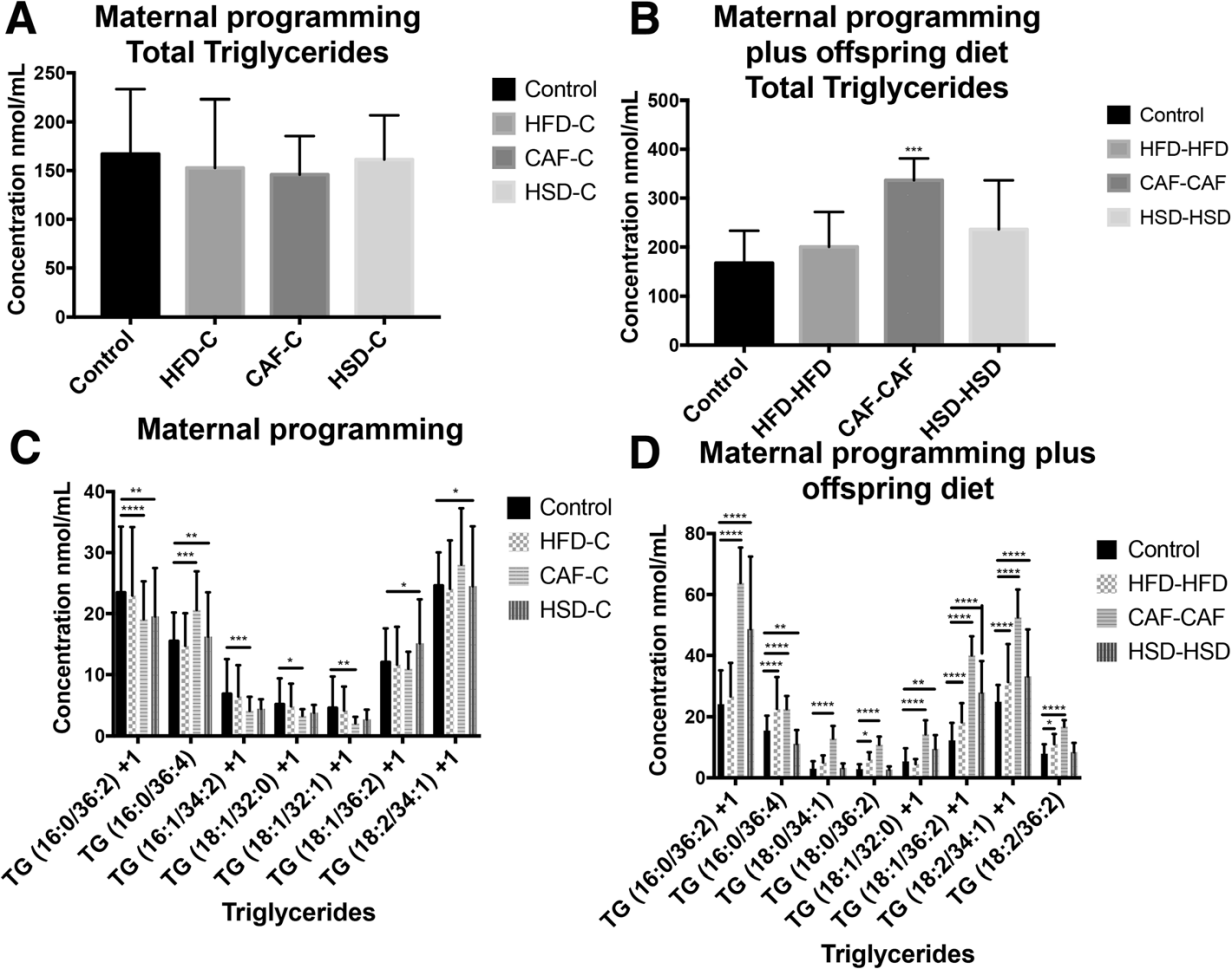
Lipidomic profile in plasma samples. Lipids were extracted from plasma samples following standard protocols and were analyzed as described in Methods. The concentration of each species was calculated using MultiQuant 3.0.1 software (AB SCIEX, Framingham, MA, USA) by relating the peak area of each species to that of the internal standard. Total plasma TG levels in maternal nutritional programming (**a**) and maternal and hypercaloric diet exposure after weaning (**b**). Selective plasma TG species in maternal nutritional programming (**c**) and maternal and hypercaloric diet exposure after weaning (**d**). Concentrations are expressed as the mean ± SEM with Chow and HFD, CAF or HSD. *n* = 10–12. *p < 0.05, **p < 0.01 and ***p < 0.001

Finally, total triglycerides (TG) and selective species were identified by lipidomic approach in the offspring. We analyzed 57 selective TG species and we found that CAF diet exposure was the most selective formula to show robust TG changes in plasma. On this context, maternal programming by HFD, CAF or HSD does not change total TG levels in offspring (Fig. 4a); however, CAF exposure during programming and after weaning in the CAF-CAF group promotes increase in total TG species (Fig. 4b). Also, maternal programming by CAF promotes increases in the selective TG species: 16: 0/36:4, 18: 2/34:1 + 1, and a decrease in 16: 0/36: 2 + 1, 16: 1/34: 2 + 1, 18: 1/32:0 + 1 and 18: 1/32:1 + 1 (Fig. 4c). Of note, CAF diet exposure after weaning increases substantially the plasma levels of the TG species: 16: 0/36:2 + 1, 16: 0/36:4, 18: 0/34:1, 18: 0/36:2, 18: 1/32:0 + 1, 18: 1/36:2 + 1, 18: 2/34:1 + 1, 18: 2/36:2 and decrease the 16: 0/36: 2 + 1, 16: 1/34: 2 + 1, 18: 1/32:0 + 1 and 18: 1/32:1 + 1 (Fig. 4d). Also, maternal programming promotes increase in 16: 0/36:4, 18: 0/36:2, 18: 2/36:2 and 16: 0/36:2 + 1, 18: 1/32:0 + 1, 18: 1/36:2 + 1 and 18: 2/34:1 + 1 for HFD and HSD, respectively; and 18: 1/36:2 + 1, 18: 2/34:1 + 1 for both programming groups (Fig. 4d).

### Nutritional programming by cafeteria diet modifies liver and white adipose tissue weight in male offspring

**Fig. 5:**
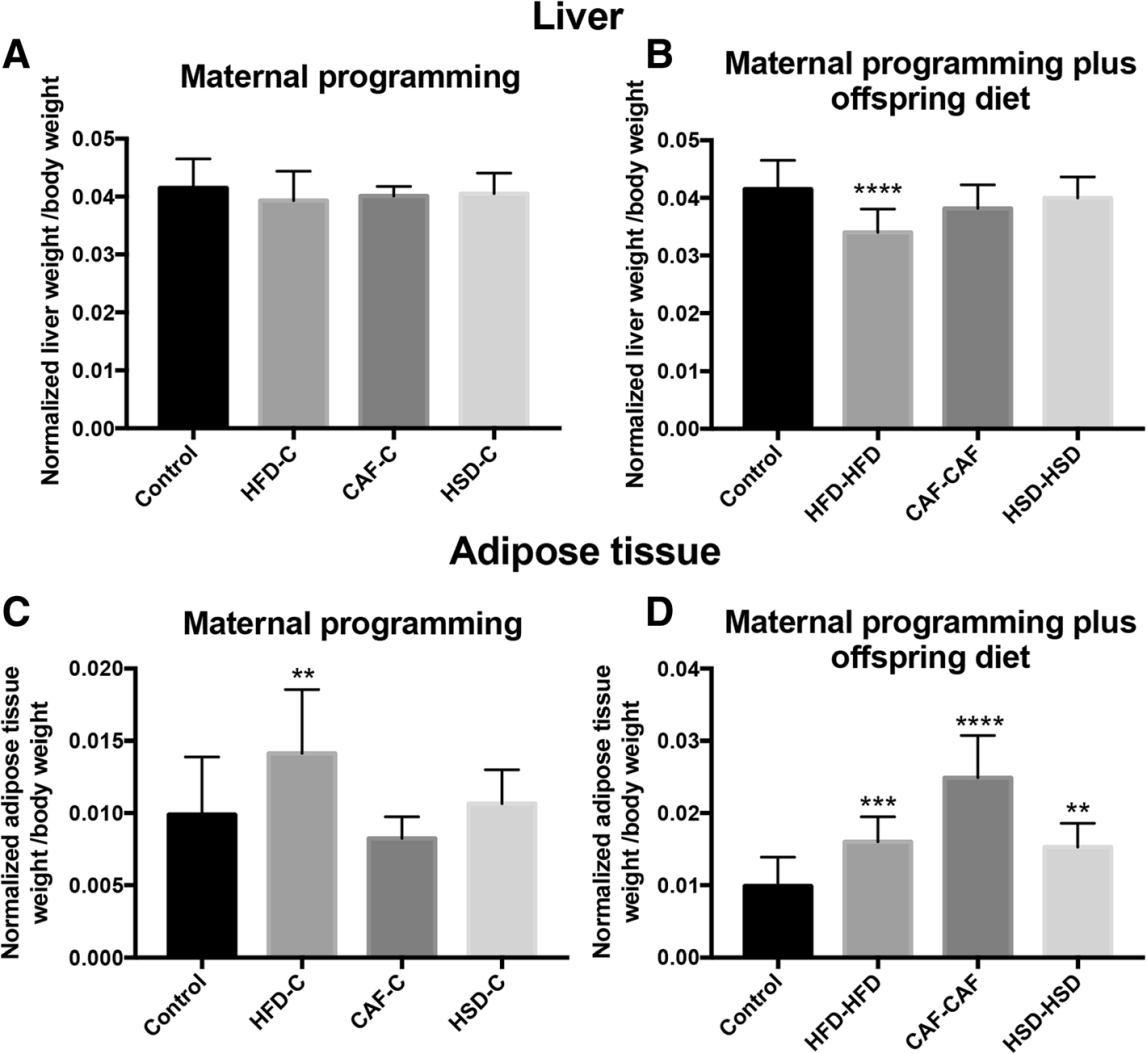
Effect of nutritional programming and offspring diet on liver and adipose tissue weight. Liver (**a**, **b**) and adipose tissue (**c**, **d**) were weighted after dissection from all groups. Data are means ± SD. *p < 0.05, **p < 0.01 and ***p < 0.001

Next, we sought to identify the effect of diets exposure during pregnancy and lactation on liver and retroperitoneal white adipose tissue weight. We identified that maternal programming by HFD, CAF or HSD exposure did not change liver weight in offspring males (Fig. 5a). On the other hand, hypercaloric surplus after weaning in the HFD-HFD group showed a decrease in liver weight when compared to control group (Fig. 5b). Also, we found that maternal metabolic programming by fat in the HFD-C group led to an increase of retroperitoneal white adipose tissue weight when it was compared to control group (Fig. 5c). Finally, hypercaloric exposure by fat or sugar intake after maternal programming, in HFD-HFD, HSD-HSD and CAF-CAF groups, increased retroperitoneal white adipose tissue weight with respect to the Chow control group (Fig. 5d).

### Hypercaloric diet exposure during pregnancy and lactation promotes hypothalamic mitochondria fusion and ER stress response in male offspring

**Fig. 6:**
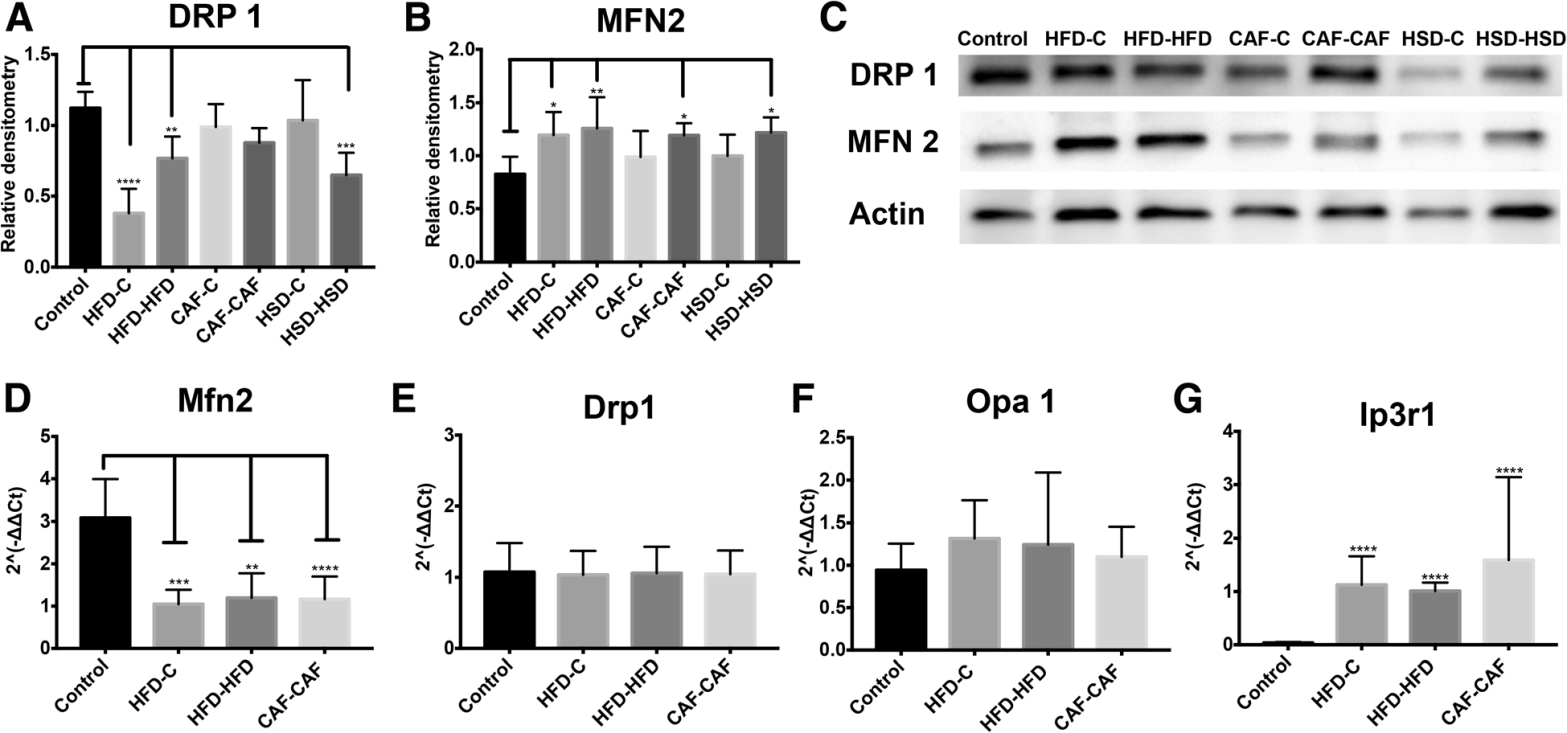
Effect of nutritional programming and offspring diet on mitochondrial dynamics in hypothalamus. Relative densitometry for protein levels of DRP1 (**a**, **c**) or MFN 2 (**b**, **c**) *n* = 8 per group. (**c**) Pictures are representative of Western Blot for DRP 1, MFN2 and Actin as loading control. mRNA expression levels of MFN2 (**d**), DRP 1 (**e**), Opa 1 (**f**) and Ip3r1 (**g**) n = 7 per group. Data are means ± SD. *p < 0.05, **p < 0.01 and ***p < 0.001

We sought to identify the effect of nutritional programming by hypercaloric diet exposure on hypothalamic mitochondria dynamics evidenced by changes in expression of proteins involved in mitochondria fusion (MFN 2, Opa 1) and/or fission (DRP 1). We found that HFD-C, HFD-HFD and HSD-HSD diets exposure led to a significant decrease in the hypothalamic DRP1 protein expression in offspring (Fig. 6a and c). By contrast, HFD-C, HFD-HFD, CAF-CAF and HSD-HSD diets showed an increase in the protein expression of MFN2 in hypothalamus (Fig. 6b and c). These data suggest that hypercaloric diet exposure during pregnancy potentially promoted positive hypothalamic mitochondrial fusion. Next, based on the results of metabolism and protein expression found in the groups of HFD-C, HFD-HFD and CAF-CAF, we quantified their mRNA expression of MFN2, Opa 1 and DRP 1 genes. We identified that HFD-C, HFD-HFD or CAF-CAF groups showed decrease expression of MFN 2 mRNA (Fig. 6d). We did not find significant differences in the mRNA expression of DRP1 and Opa 1 genes (Fig. 6e and f). We also analyzed the expression of Ip3r1, which is related to calcium homeostasis between ER and mitochondria and it has been reported upregulated in obesity or lipotoxicity [21]. Offspring fed with HFD or CAF after maternal programming (HFD-HFD and CAF-CAF) increased the mRNA expression of Ip3r1 gene (Fig. 6g).

**Fig. 7:**
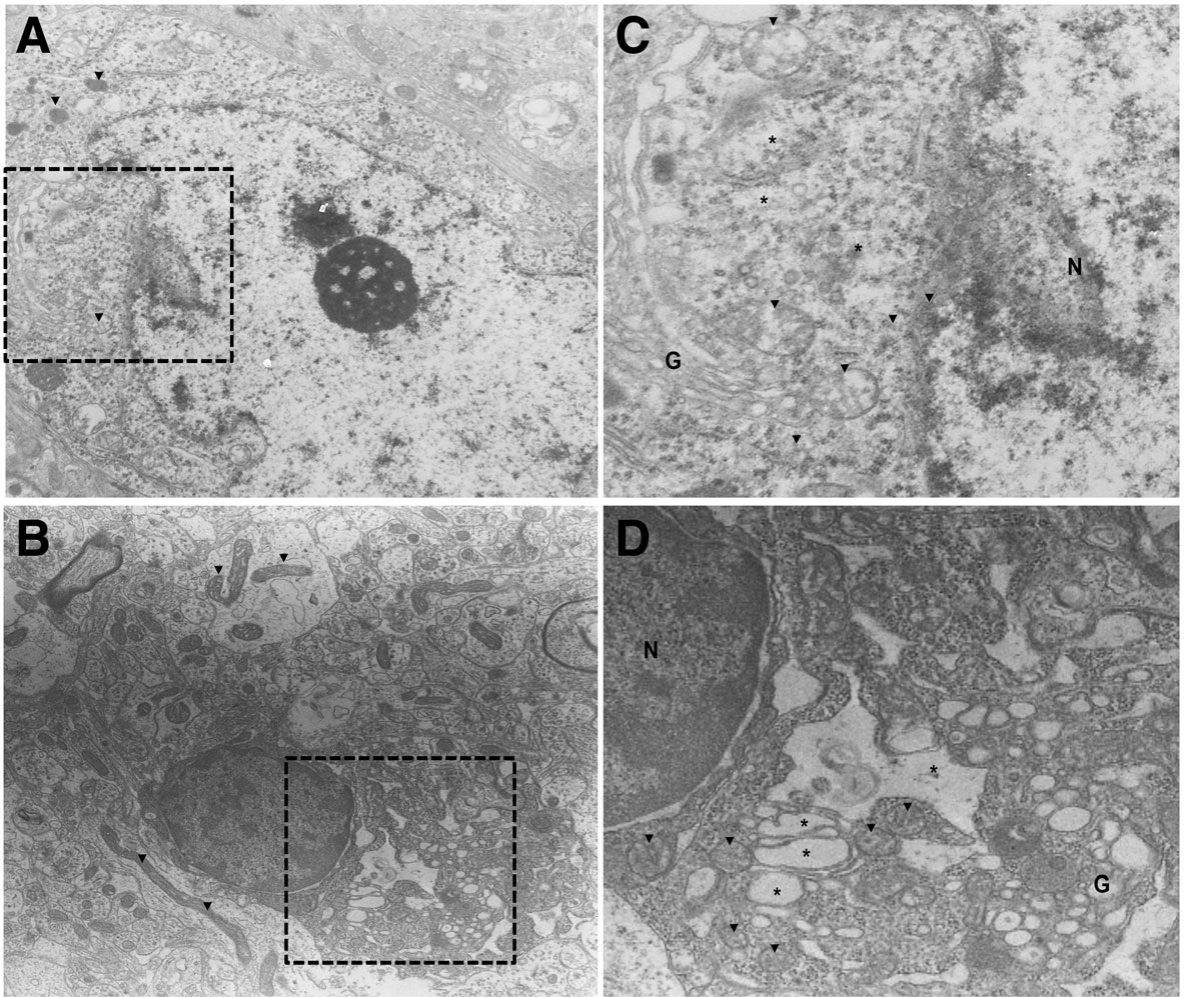
Effect of nutritional programming of CAF diet plus offspring CAF diet on hypothalamus ultrastructure. **a**) Control, **b**) CAF-CAF groups. Note that CAF-CAF diet promote appearance of bigger mitochondria and mitochondria-ER interactions in CAF-CAF (**d**, 3000×) in compare with Control (**c**, 3000×). Unstacked and extremely distended endoplasmic reticulum cisternae rims extended (* asterisk) around mitochondria (▼ arrow head) was observed in CAF-CAF group. N, nucleus; G, Golgi apparatus

Additionally, we identified morphological alterations in ER and mitochondria in CAF-CAF, and Control experimental groups by using TEM. We chose CAF-CAF because is one of the groups with the greatest difference in metabolism and mitochondrial dynamics. We observed that nutritional programming and offspring intake of hypercaloric diet after weaning in the CAF-CAF group promotes evident hypothalamic ER disorganization, with unstacked cisternae rims, which were extremely distended and surrounding mitochondria and bigger mitochondria (Fig. 7b and d). These results indicated an increase in the ER-mitochondria contacts and confirm our previous findings at molecular level showing that CAF-CAF intake lead to mitochondrial fusion and ER dysfunction.

### Lipotoxicity of saturated fatty palmitic acid induced decrease of ER signal, mitochondrial mass, membrane potential (ΔΨm) and mitochondrial calcium overload

**Fig. 8:**
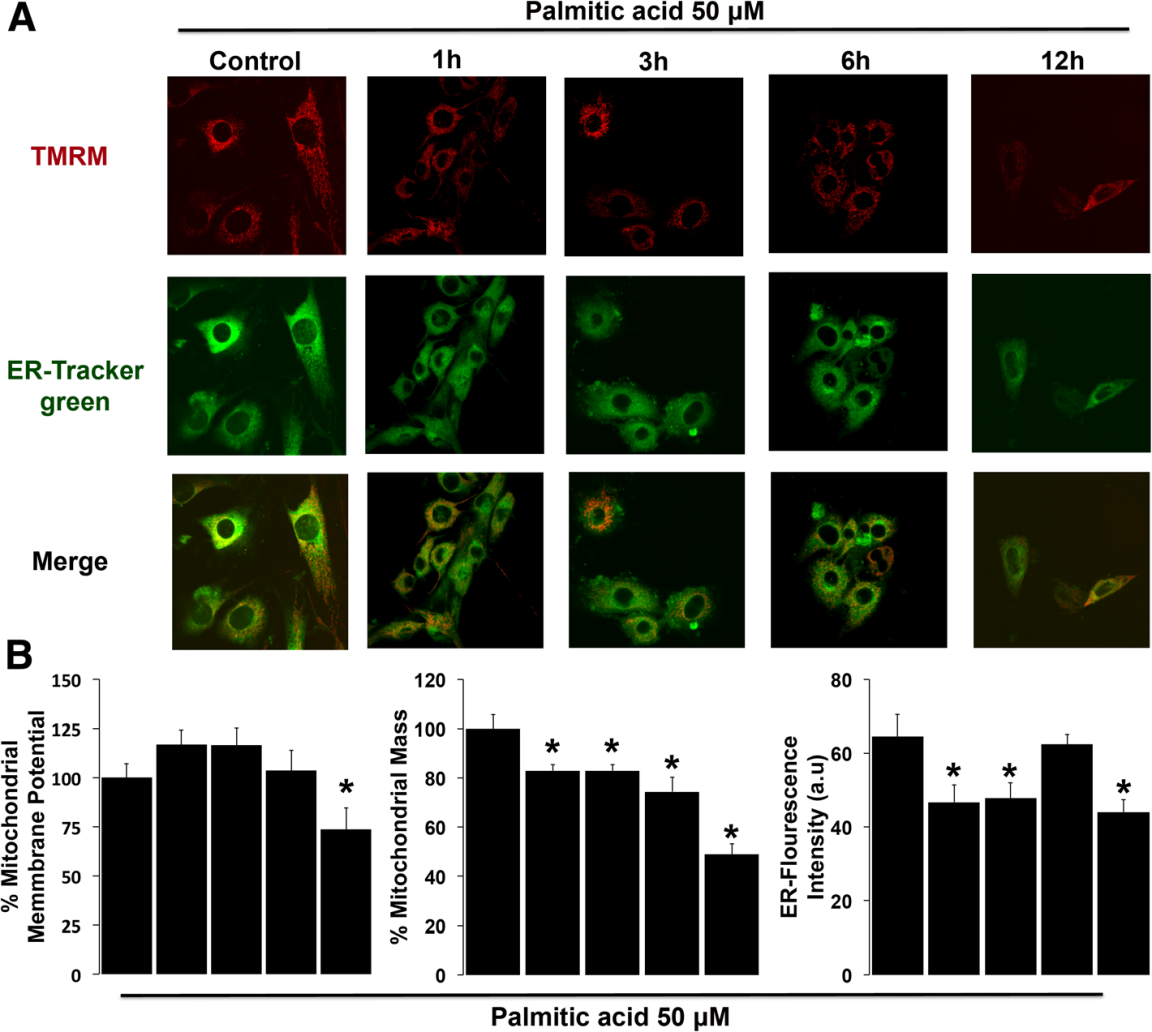
Lipotoxicity of palmitic acid induced decrease in mitochondrial mass, membrane potential (ΔΨm) and in ER signal. (**a**) Representative confocal microscopy images of ΔΨm, which was measured by the retention of TMRM (red), and ER-Tracker Green to define the cytosol. (**b**) Quantification of mitochondrial membrane potential. Mitochondrial mass, which was calculated from the images stained with TMRM and ER-Tracker Green. ER-Tracker Green fluorescence intensity. Data are mean ± SEM, and values are from three independent experiments. *p < 0.01 compared to control group. Scale bar = 10 μm

Based on our previous results showing that hypercaloric diet exposure promoted an increase in mitochondrial fusion and in endoplasmic reticulum Ip3r1 mRNA expression in hypothalamus, and, that CAF diet exposure after weaning (CAF-CAF group) increases mitochondrial fusion and ER contacts in offspring, we sought to determine the physiological role of ER-mitochondria. It has been reported that positive energy balance during obesity promotes enhanced ER-mitochondria contacts, modulated mitochondria physiology by calcium fluxes [21]. We analyzed in an in vitro system whether the lipotoxicity insult induced by the saturated fatty palmitic acid modulates calcium fluxes from ER to mitochondria triggering mitochondrial dysfunction. Hypothalamic cells were loaded with TMRM, treated with the indicated conditions and were quantified as described using confocal microscopy to determine changes in ΔΨm (Fig. 8a). Palmitic acid stimulation during 1 and 3 h to hypothalamic cells induced an increase of ΔΨm, however after 6 h of treatment we found a decrease of ΔΨm, this effect was exacerbated at 12 h (Fig. 8b), showing that palmitic acid induces mitochondrial dysfunction.

To assess mitochondrial mass and ER function, cells were co-loaded with TMRM to label mitochondria and with ER-Tracker Green, which aid to label the cytosol, allowing measurement of the volume occupancy of the mitochondrial network within the dimensions of the cytosol and ER (Fig. 8b). At 1, 3 and 6 h after palmitic acid treatment we identified a significant decrease in mitochondrial mass (Fig. 8b), which was more evident at 12 h of treatment with palmitic acid. Similarly, we observed a decrease in the ER-Tracker Green fluorescence intensity when hypothalamic cells were treated for 1 and 3 h and an increase at 6 h. However, at 12 h we observed again a decrease similar to the one observed at 1 and 3 h, which indicates ER stress (Fig. 8b). These results showed that lipotoxic insult promotes time-dependent hypothalamic mitochondria and ER dysfunction.

**Fig. 9:**
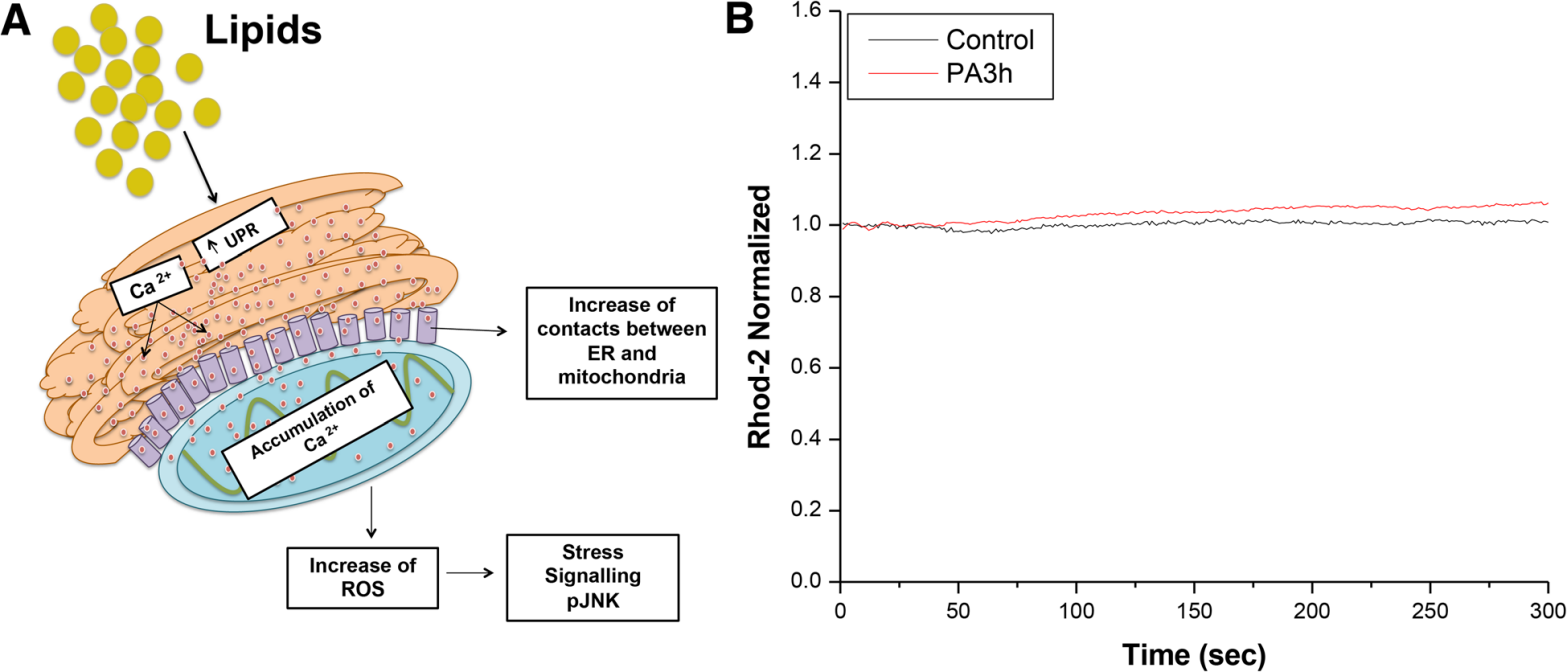
Lipotoxicity of palmitic acid induced changes in mitochondrial Ca^2+^ levels. **a**) Hypothetical model of calcium over flux from ER to mitochondria and metabolic complications during lipotoxicity. **b**) Mitochondrial Ca^2+^ after 3 h of treatment with palmitic acid and the normalised values of Rhod-2 AM fluorescence are shown in the histogram. Data are mean ± SEM, and values are from three independent experiments

Next, we sought to identify time-dependent changes in mitochondrial Ca^2+^ levels during lipotoxicity of saturated fatty palmitic acid stimulation. After 3 h of treatment with palmitic acid, cells were incubated with the mitochondrial Ca^2+^ indicator Rhod-2 AM. Palmitic acid-treated cells exhibited a slight increase in Rhod-2 fluorescence, indicating elevated mitochondrial Ca^2+^ levels (Fig. 9).

## Discussion

Obesity and maternal overnutrition during pregnancy lead to several metabolic changes that trigger chronic related diseases including Type 2 diabetes mellitus (T2MD) in offspring [7, 13, 14, 20]. We sought to identify whether hypercaloric diets exposure including HFD, CAF and HSD, during pregnancy and lactation selectively modulates body metabolic parameters and hypothalamic mitochondrial dynamics in male offspring after weaning. We compared the hypercaloric formula to standard chow diet formula which has low fat or carbohydrates percentage. Diet might be the major driving force behind changes in parameters as microbiota [32] and also CAF diet is considered a robust model of metabolic syndrome when it is compared to HFD [33]. In this context, we identified that selective maternal overnutrition and hypercaloric exposure in male offspring after weaning using the HFD or CAF diets led to body weight alterations and increase in glucose, insulin, leptin and TG levels. Also, we found that HFD maternal programming is effective to promote hypothalamic mitochondria fusion as well as the consumption of the HFD, CAF and HSD diets by the offspring after maternal programming.

Our initial results showed that dams subjected to CAF formula weight significantly less before delivery when compare to control, HFD or HSD diets. In offspring, we found that CAF diet decreased birthweight and whereas HSD increased birthweight. It is known that maternal overnutrition and obesity in females might impact survival and normal development in offspring [13, 14] and also increase body weight in rats and in non-human primates with diets like HSD [31, 34, 35]. In fact, there is evidence that maternal HFD exposure in rats does not promote changes in offspring body weight at birth [36]. A recent meta-analysis study showed that maternal HFD increased birth weight in male mice, and there was a trend in male rats to show low birth weight [37]. We hypothesized that no correlation from our results compared with previous studies might be potentially due to the murine strain, age and time of diet exposure, as reported previously [36, 38, 39].

Next, we identified the effect of nutritional programming after weaning in body weight of offspring. At this stage, we modulated hypercaloric surplus in offspring by exposure to Chow control diet or keep them with the hypercaloric mother’s diet. We found that maternal HFD exposure (HFD-C group) increases body weight whereas HFD-HFD and CAF-CAF decrease body weight at 5th–6th and 4th–7th weeks of age respectively, compared with Control group. Also, CAF exposure after maternal programming (CAF-CAF group) had an increase in food intake at 6th week. Supporting our findings, CAF formula decreases rate of growth shortly after its exposure [40], and on the other hand, maternal programming by HFD exposure leads to body weight increase in mice offspring [31] tentatively associated to hyperphagia from 5 to 6 weeks of age [41, 42]. In this context, we did not find difference between HFD-C and Control group in food intake; however, the HFD-HFD group where dams and offspring were fed HFD displayed a substantial decrease in food intake, disruption in food efficiency and body weight versus Control group. These results suggest that developmental nutritional programming might set up a threshold to increase the susceptibility to metabolic dysfunction. In any case, it would be potentially important to determine food intake at adulthood to define time dependent effects of hypercaloric exposure before and after weaning.

Our data showed that maternal programming by CAF-C decreases plasma glucose levels during the GTT analysis, whereas the HFD- C group showed an increase in plasma glucose during ITT test. These results partially agree with previous reports showing maternal HFD effect on glucose increase at 6 weeks of age in offspring [31, 43] and with the fact that maternal HFD reduced insulin tolerance [42]. In addition, CAF diet exposure increases plasma glucose levels in male and female offspring at the age of 3 months [44]. No correlations between our results and the reported by Desai et al. 2014 and Melo et al. 2014, might be related to the age of the subjects, 24 weeks versus 8 weeks from our studies [31, 43]. However, we do report that nutritional programming by HFD or CAF decrease insulin sensitivity and enhance glucose metabolism during the ITT and GTT, respectively. Also, hypercaloric stimuli by HDF or CAF exposure after maternal programming lead to an increase in plasma insulin, leptin and TG lipid species levels compared to control, which correlates with an increase in retroperitoneal white adipose tissue weight in these groups as well as a decrease in liver weight in HFD-HFD and CAF-CAF. These results agree with previous data reported where increased leptin and insulin concentrations were found in young rats and obese rats on HFD [45–47]. Our data also show that compare to HFD and HSD which also show glucose, leptin and insulin deregulation, the consumption of CAF diet after weaning in offspring seems to be the most significant nutritional formula to promote changes in total TG plasma concentrations and in selective TG species. The change in the profiles of triglycerides, ceramides and dhCer have been linked to insulin resistance and obesity [48] and alterations in the expression of ceramide synthase and dihydroceramide desaturase increasing lipotoxicity [49, 50]. Additionally, our data correlate with negative effects on normal organ weight during hypercaloric nutritional programming [31]. Overall, we speculate that initial metabolic, hormonal and organ weight alterations in offspring at 8 weeks, found in our experimental models, might compromise basal metabolic settings and potentially increase the susceptibility to glucose imbalance in the adulthood.

Metabolic programming during maternal overnutrition and obesity leads to ER stress activation [13, 14, 31, 51, 52], and mitochondrial dysfunction in oocytes and embryos [14, 20], and impairs hypothalamic glucose metabolism in male offspring [53]. It is known that mitochondrial dynamics is regulated by cellular bioenergetic demands [54]. For instance, during high energy demand mitochondrial fusion results in extended mitochondrial networks which provides advantage to cell homeostasis; however, disruption of mitochondrial fusion has been shown to result in energy failure and mitochondrial dysfunction [54]. Here we added new evidence by showing that HFD-C, HFD-HFD, CAF-CAF and HSD-HSD exposure decrease the mitochondrial fission marker DRP1 and increase the fusion MFN 2 protein marker expression in the hypothalamus of offspring. Of importance, mitochondrial dysfunction shows a lower expression of the mitochondrial fusion marker protein OPA1, which correlates with increased expression of the mitochondrial fission marker protein DRP1 [54]. In our case, we found a negative feedback caused by maternal programming and offspring diet that increase MFN 2 and decrease in DRP 1 protein levels, which potentially suggest positive mitochondria fusion. We have not found correlation between mRNA expression and total protein levels of MFN 2 and DRP1, our observations by TEM suggest an enhance in mitochondria-ER contacts in the offspring of CAF-C group and positive hypothalamic mitochondria fusion, bigger mitochondria and enhance mitochondria-ER contacts in the CAF-CAF group. Hypothalamic DRP1 regulates ROS signaling in glucose sensing [55] and also modulates leptin sensitivity in POMC neurons [56]. DRP1 ablation leads to higher ROS production and dysfunctional mitochondria [57]. On the other hand, others and we have reported up regulation of hypothalamic MFN2 protein levels of obese mice [21, 22], and defective MFN2-expression in POMC or Agrp neurons promotes or prevents obesity in a rodent obese model, respectively [23, 24]. Also, obesity leads to changes in mitochondrial morphology in liver and increases ER-mitochondria junctions [21], suggesting that MFN2 regulation might influence susceptibility to gain more weight during growth, as shown previously and metabolic dysfunction [9, 58]. It would be relevant to determine selective changes in mitochondrial dynamics in adulthood and identify whether there is a potential transgenerational effect, as has been reported in other animal models [20]. Overall, our data propose that hypercaloric nutritional programing leads to exacerbation of mitochondria fusion in hypothalamus, which correlates, with metabolic compromise in offspring.

Finally, we tested the hypothesis whether increase in mitochondria fusion and ER-mitochondria contacts are potential negative modulators of mitochondria metabolism. It has reported that the onset of mitochondrial dysfunction is secondary to ER stress and Ca^2+^ release [59, 60]. We initially identified that HFD-C decreases expression of Hspa5 and HFD-C, HFD-HFD and CAF-CAF manipulation increases Ip3r1 expression, two ER calcium markers. We found that palmitic acid stimulation to hypothalamic cells leads to ER stress, which correlates with a decrease in both mitochondrial membrane potential and mitochondrial mass at 6 h. Of note, palmitic acid stimulation increases mitochondrial Ca^2+^ levels at earlier time-course such as 3 h after treatment. This supports the hypothesis that lipotoxic stimulation with palmitic acid promotes initial Ca^2+^ release from ER leaking to mitochondria matrix and tentatively promote mitochondrial dysfunction. These results also agree with the increase of the expression of Ip3r1 in maternal hypercaloric diets, given its role on Ca^2+^ flux from ER to mitochondria [21, 59, 61].

## Conclusions

Maternal programming by HFD, CAF or HSD increase insulin and leptin plasma levels, respectively, however, HFD maternal programming exposure is potentially effective in promoting hypothalamic mitochondrial fusion and ER stress in the offspring. Positive mitochondria fusion is replicated in male offspring programmed by HFD, CAF and HSD and exposure to HFD, CAF and HSD after weaning, which correlate with failure in glucose, leptin and insulin sensitivity and fat accumulation for the HFD and CAF nutrient exposure. We suggest that lipotoxic insults related to lipid overload might lead to mitochondrial dysfunction linked to calcium overload by the ER-mitochondria crosstalk.

## Abbreviations

C: Control
CAF: Cafeteria diet
DIO: Diet induced obesity
DRP1: Dynamin-related protein 1
ER: Endoplasmic reticulum
GTT: Glucose tolerance test
HFD: High Fat Diet
HSD: High Sugar Diet
Ip3R1: Inositol 1,4,5-Trisphosphate Receptor Type 1
ITT: Insulin tolerance test
MFN2: Mitofusin 2
mRNA: Messenger RNA
OPA1: Optic Atrophy type 1
POMC: Proopiomelanocortin
ROS: Reactive Oxigen Species
T2DM: Type 2 diabetes mellitus
TEM: Transmission electron microscopy
TMRM: Tetramethylrhodamine
